# λ-Carrageenan promotes plant growth in banana via enhancement of cellular metabolism, nutrient uptake, and cellular homeostasis

**DOI:** 10.1038/s41598-022-21909-7

**Published:** 2022-11-16

**Authors:** Kah-Lok Thye, Wan Muhamad Asrul Nizam Wan Abdullah, Zetty Norhana Balia Yusof, Chien-Yeong Wee, Janna Ong-Abdullah, Jiun-Yan Loh, Wan-Hee Cheng, Dhilia Udie Lamasudin, Kok-Song Lai

**Affiliations:** 1 Department of Cell and Molecular Biology, Faculty of Biotechnology and Biomolecular Sciences, Universiti Putra Malaysia, 43400 Serdang, Selangor Malaysia; 2 Department of Biochemistry, Faculty of Biotechnology and Biomolecular Sciences, Universiti Putra Malaysia, 43400 Serdang, Selangor Malaysia; 3 Biotechnology and Nanotechnology Research Centre, Malaysian Agricultural Research and Development Institute, 43400 Serdang, Selangor Malaysia; 4 Centre of Research for Advanced Aquaculture (CORAA), UCSI University, 56000 Cheras, Kuala Lumpur Malaysia; 5 Faculty of Health and Life Sciences, INTI International University, Persiaran Perdana BBN, Putra Nilai, 71800 Nilai, Negeri Sembilan Malaysia; 6 Health Sciences Division, Abu Dhabi Women’s College, Higher Colleges of Technology, 41012 Abu Dhabi, United Arab Emirates

**Keywords:** Plant sciences, Plant development, Plant physiology

## Abstract

Banana (*Musa acuminata*) is an important fruit crop and source of income for various countries, including Malaysia. To date, current agrochemical practice has become a disputable issue due to its detrimental effect on the environment. λ-carrageenan, a natural polysaccharide extracted from edible red seaweed, has been claimed to be a potential plant growth stimulator. Hence, the present study investigates the effects of λ-carrageenan on plant growth using *Musa acuminata* cv. Berangan (AAA). Vegetative growth such as plant height, root length, pseudostem diameter, and fresh weight was improved significantly in λ-carrageenan-treated banana plants at an optimum concentration of 750 ppm. Enhancement of root structure was also observed in optimum λ-carrageenan treatment, facilitating nutrients uptake in banana plants. Further biochemical assays and gene expression analysis revealed that the increment in growth performance was consistent with the increase of chlorophyll content, protein content, and phenolic content, suggesting that λ-carrageenan increases photosynthesis rate, protein biosynthesis, and secondary metabolites biosynthesis which eventually stimulate growth. Besides, λ-carrageenan at optimum concentration also increased catalase and peroxidase activities, which led to a significant reduction in hydrogen peroxide and malondialdehyde, maintaining cellular homeostasis in banana plants. Altogether, λ-carrageenan at optimum concentration improves the growth of banana plants via inducing metabolic processes, enhancing nutrient uptake, and regulation of cell homeostasis. Further investigations are needed to evaluate the effectiveness of λ-carrageenan on banana plants under field conditions.

## Introduction

*Musa acuminata*, commonly known as banana, is the fourth most important food crop after rice, wheat, and maize^1^. Banana is the largest herbaceous flowering plant from the family of Musaceae, which grows in tropical and subtropical regions. The global banana consumption was recorded at 125 million tons in 2016 and is expected to rise to 150 million tons by 2025^2^. Hence, to meet the increasing global demand, banana production must be increased.

To date, banana production is highly affected by challenges such as climate change, limiting agricultural lands, poor crop management practices, as well as pests and diseases. For example, global warming and decrease in rainfall had led to an insufficient water supply that reduced the banana yield and production^3^. Besides, the increase of urban land expansion has caused a drastic reduction of suitable agricultural lands for banana cultivation. Moreover, commercial bananas are highly susceptible to pests and diseases like Fusarium wilt^4^, banana bunchy top virus^5^, and banana weevil^6^. Currently, nitrogen, phosphorus, and potassium (N:P:K) fertilizer has been commonly used as the main nutrient resources to improve banana growth and yield. However, improper usage of N:P:K fertilizer may cause harmful effects on crop growth^7^. Overuse of fertilizer would damage the crop and cause environmental pollution^8^. The excessive fertilizer will be washed away into water sources such as rivers and ponds, causing pollution and even the death of aquatic animals. Hence, an alternative and more environmentally friendly agricultural strategy is needed to boost banana growth.

Previously, plant elicitors, either biotic or abiotic, had been employed to stimulate plant growth for better yield production^9^. This included the usage of elicitors such as carrageenan, chitosan, β-Aminobutyric acid, lignosulfonate and beneficial micro-organisms^9–11^. Carrageenan is a naturally occurring high molecular weight polysaccharide, which is extracted from edible red seaweed (Rhodophyta)^11^. It can be classified into several forms based on the degree of sulfation, with iota (ι), kappa (κ), and lambda (λ) being the most commercially important^12^. As a natural polymer, it has been commonly used in many areas, such as the food industry, cosmetics, and pharmaceuticals, due to its eco-friendly nature^12^. In recent years, carrageenan has also been reported to promote plant growth and defense response against pathogens in several plant species. It successfully enhanced the growth of tobacco^13^, chickpea^14^, maize^14^, *Pinus radiata*^15^, and *Eucalyptus globulus*^16^. Although its underlying plant growth-promoting response remains largely unknown, it has been hypothesized that carrageenan tends to promote plant growth by enhancing photosynthesis^13,17^. Besides, González et al.^17^ proposed that carrageenan may improve plant growth through basal metabolism enhancement. Apart from its plant growth-promoting effect, carrageenan could also elicit defense response against fungi, bacteria, virus, and insect^11,18^. Nonetheless, carrageenan could serve as a potential dual-functional bio-fertilizer for plant growth enhancement and plant defense response against pathogens, whereby it could be an environmentally safer approach for fertilizing plants^14^. Among the carrageenans, λ-carrageenan showed a higher degree of sulfation (32% to 39%) which may induce higher eliciting activity^18,19^. Mercier et al.^18^ also demonstrated that λ-carrageenan worked in a dose-dependent manner. Hence, proper concentration optimisation is needed for maximizing the usage of λ-carrageenan in agriculture.

To the best of our knowledge, the use of λ-carrageenan to enhance the growth of the banana plant has not been reported. Besides, the underlying growth-promoting response of λ-carrageenan remains largely unknown. Hence, the objective of this study is to evaluate the effects of λ-carrageenan on the growth of *Musa acuminata* cv. Berangan (AAA). In addition, gene expression profiling, biochemical assays, and nutrient analysis were also performed to shed light on the underlying plant growth-promoting mechanism of λ-carrageenan. Ultimately, it is our goal to enhance the growth performance of the banana plant and increase its production via the application of λ-carrageenan.

## Results

### Effect of different λ-carrageenan concentrations on banana plants growth

**Figure 1:**
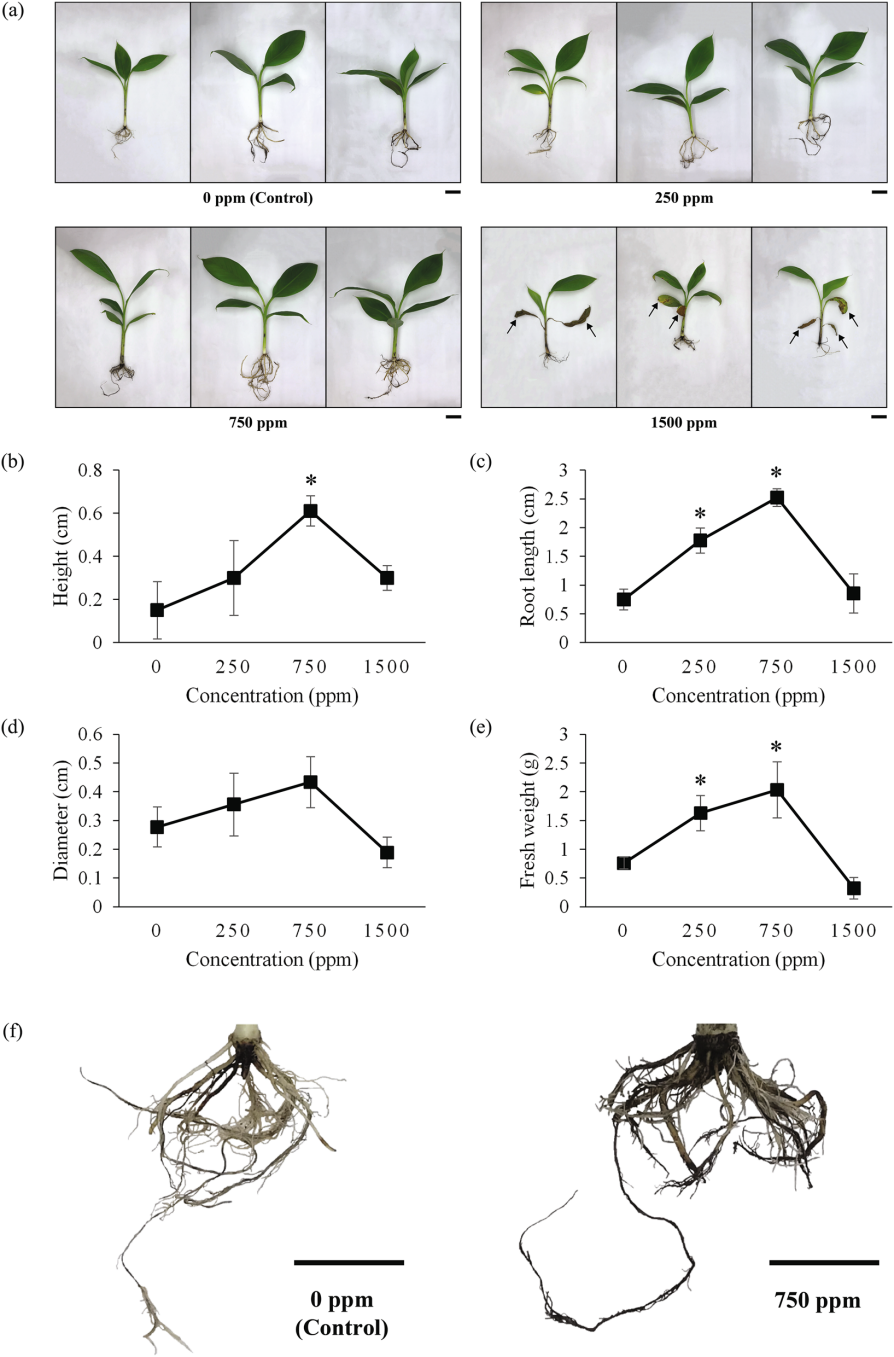
Effects of different λ-carrageenan concentrations on growth performance of banana plants. (**a**) Representative images of λ-carrageenan-treated banana plants for each treatment. (**b**) Average increment in plant height. (**c**) Average increment in root length. (**d**) Average increment in pseudostem diameter. (**e**) Average increment in fresh weight. (**f**) Enhancement on root structures of banana plants treated with optimum concentration (750 ppm) of λ-carrageenan compared to control. Scale bars represent 5 cm. Arrows show yellowing leaves. Data represents mean values (± SE) of three independent experiments. Asterisk (*) indicates statistically significant difference between the control and λ-carrageenan-treated samples as determined by Dunnett’s test at P < 0.05.

Optimisation of λ-carrageenan concentration on banana plants were carried out to assess the effect of λ-carrageenan on the growth performance of banana plants. As shown in Fig. 1, a significant increase in growth performance was observed in banana plants treated with 250 ppm and 750 ppm λ-carrageenan, compared to control plants. The plants treated with 750 ppm λ-carrageenan recorded the highest increment in height (0.611 ± 0.07 cm) (Fig. 1b), root length (2.52 ± 0.15 cm) (Fig. 1c), pseudostem diameter (0.43 ± 0.09 cm) (Fig. 1d), and fresh weight (2.03 ± 0.49 g) (Fig. 1e). A significant enhancement of root structure was also observed in banana plants treated with 750 ppm treatment, compared to the control plants (Fig. 1f). In contrast, the plant growth performance decreases beyond the concentration of 750 ppm. The plant treated with λ-carrageenan at 1500 ppm displayed slight increment in plant height (0.3 ± 0.06 cm) (Fig. 1b) and root length (0.86 ± 0.34 cm) (Fig. 1c) but showed a decrement in pseudostem diameter (0.19 ± 0.05 cm) (Fig. 1d) and fresh weight (0.32 ± 0.19 g) (Fig. 1e). Moreover, yellowing of leaves was observed in banana plants treated with 1500 ppm of λ-carrageenan (Fig. 1a).

### Gene expression profiling of banana plants in response to λ-carrageenan treatment

**Figure 2:**
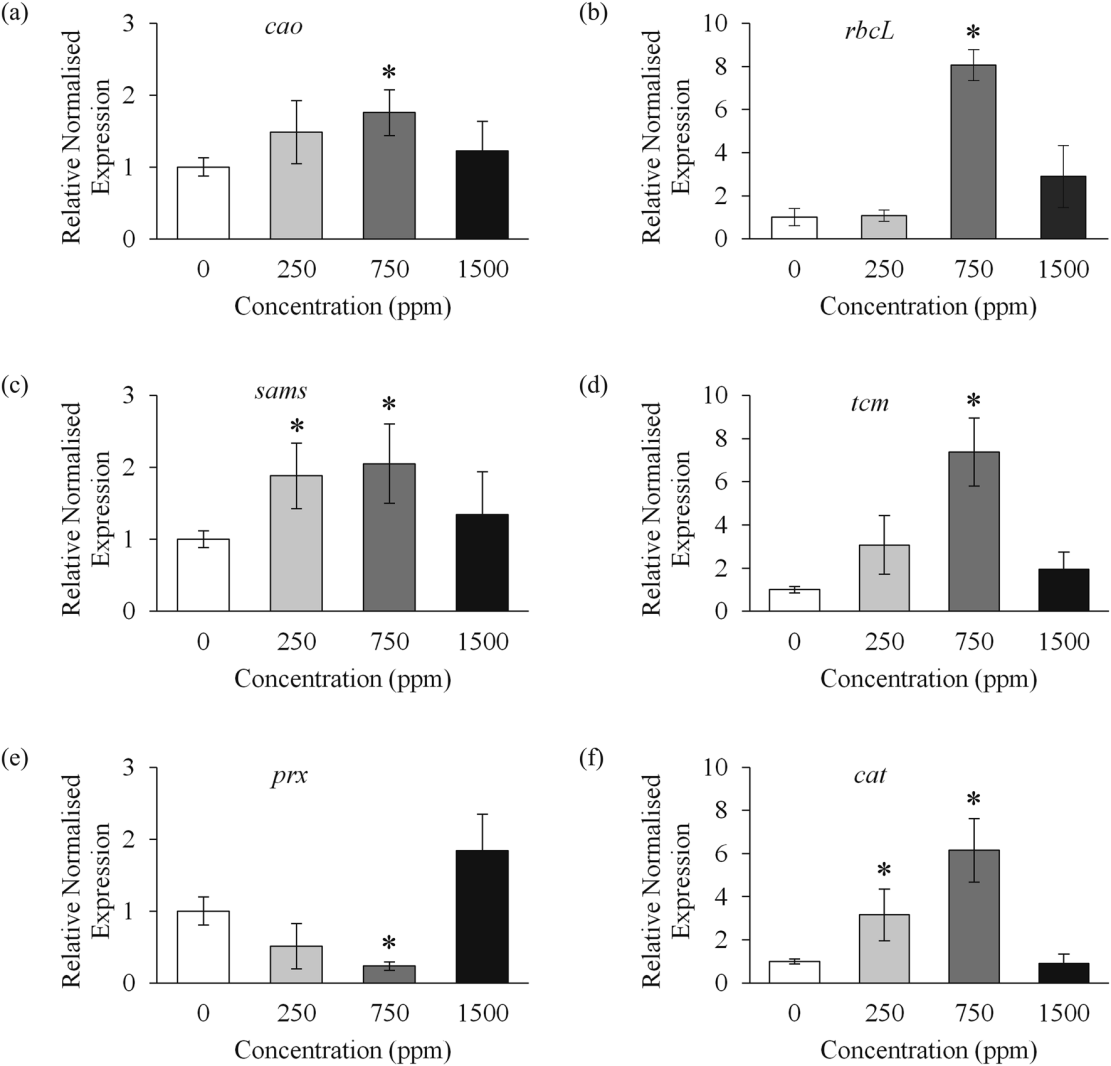
Expression patterns of *cao* (**a**), *rbcL* (**b**), *sams* (**c**), *tcm* (**d**), *prx* (**e**) and *cat* (**f**) genes in banana plants treated with different λ-carrageenan concentrations. Results are presented as differential relative transcript abundance. Bars represent mean values (± SE) of three independent experiments. Asterisk (*) indicates statistically significant difference between the control and λ-carrageenan-treated samples as determined by Dunnett’s test at P < 0.05.

To study whether λ-carrageenan affects the expression of genes involved in growth promotion, six different target genes involved for respective biochemical assays were examined using qRT-PCR, namely *chlorophyllide a oxygenase* (*cao*), *ribulose-1,5-bisphosphate carboxylase* (*rbcL*), *S-adenosylmethionine synthase* (*sams*), *trans-cinnamate 4-monooxygenase* (*tcm*), Class III *peroxidase* (*prx*) and *catalase* (*cat*). As shown in Fig. 2, the application of λ-carrageenan significantly changes the relative expression of all these genes, compared to control. The mRNA transcripts level of *cao*, *rbcL*, *sams* and *tcm* were upregulated in all banana plants treated with λ-carrageenan (Fig. 2a–d). A significant increase of *cao* (1.76-fold), *rbcL* (8.07-fold), *sams* (2.05-fold), and *tcm* (7.38-fold) transcripts was recorded in banana plants treated with 750 ppm of λ-carrageenan. Similarly, a significant increment of *sams* gene expression was also found in 250 ppm treatment (1.88-fold). However, downregulation of Class III *prx* gene expression was detected in 250 ppm (1.95-fold) and 750 ppm (4.28-fold) λ-carrageenan treatment (Fig. 2e). In 1500 ppm treatment, the expression level of *prx* transcript was increased up to 1.84 folds. On the other hand, the expression of *cat* transcripts were significantly induced in 250 ppm (3.16-fold) and 750 ppm treatment (6.15-fold) (Fig. 2f).

### λ-Carrageenan enhances photosynthetic activities in banana plants

**Figure 3:**
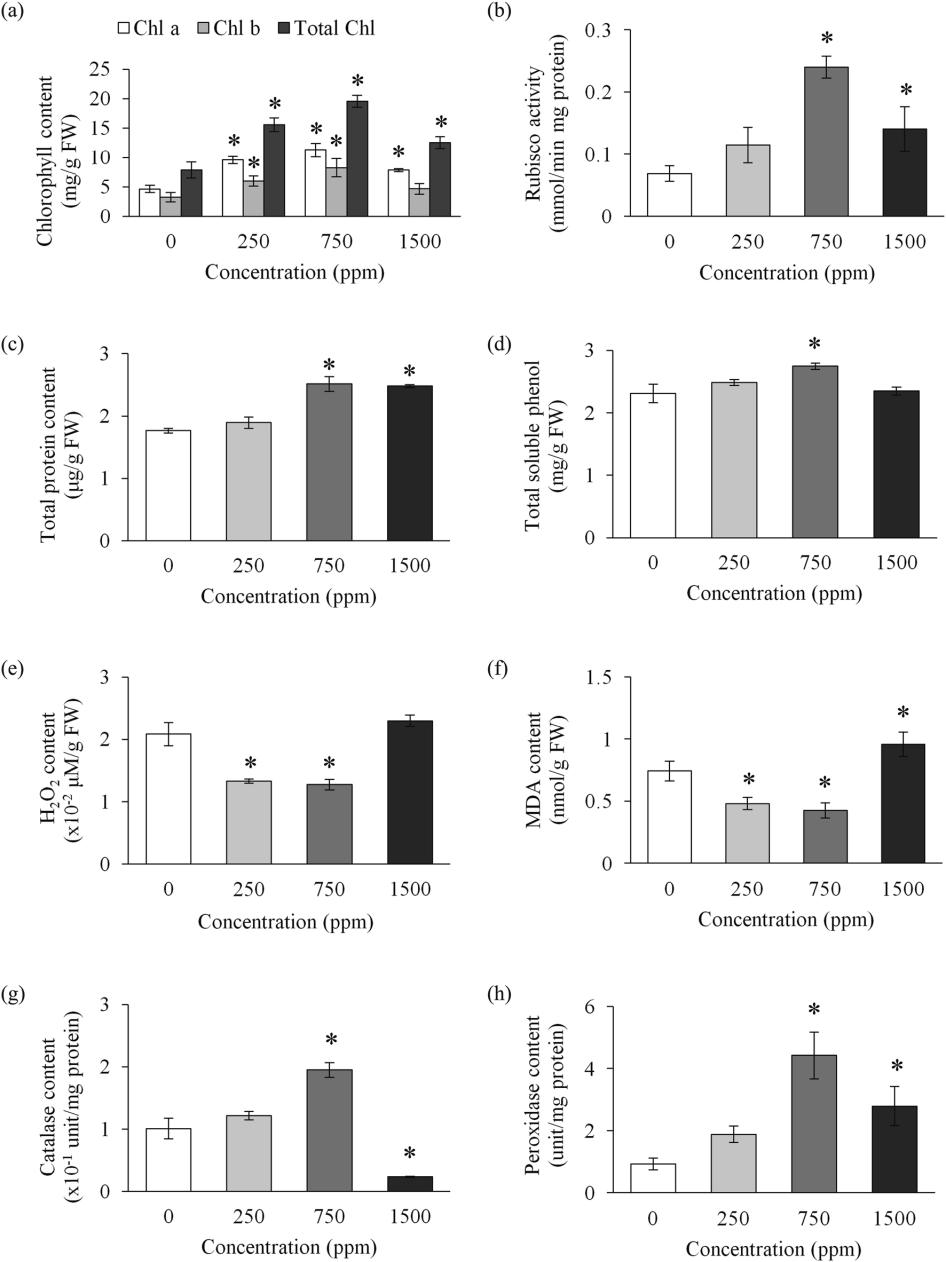
Effects of different λ-carrageenan concentrations on biochemical activities associated with growth promotion. (**a**) Chlorophyll content. (**b**) Rubisco activity. (**c**) Total protein content. (**d**) Total soluble phenol content. (**e**) Total hydrogen peroxide content. (**f**) Malondialdehyde content. (**g**) Total catalase content. (**h**) Total peroxidase content. Bars represent mean values (± SE) of three independent experiments. Asterisk (*) indicates statistically significant difference between the control and λ-carrageenan-treated samples as determined by Dunnett’s test at P < 0.05.

All banana plants treated with λ-carrageenan showed an increase in chlorophyll *a* and *b* contents, as well as total chlorophyll content (Fig. 3a). The highest levels of chlorophyll *a* and chlorophyll *b* contents were observed in 750 ppm λ-carrageenan treatment (11.25 ± 1.13 mg/g FW and 8.27 ± 1.54 mg/g FW, respectively) (Fig. 3a). In comparison to the control plants (7.86 ± 1.37 mg/g FW), plants treated with 750 ppm λ-carrageenan showed an increase in total chlorophyll content (19.52 ± 1.02 mg/g FW), which paralleled the vegetative growth observed in Fig. 1.

In addition, all banana plants treated with λ-carrageenan also showed higher ribulose-1,5-bisphosphate carboxylase/oxygenase (rubisco) activity as compared to control plants. Among the λ-carrageenan treatments, the banana plants treated with 750 ppm recorded the highest rubisco activity (0.24 ± 0.017 mmol/min mg protein), compared to the control (0.07 ± 0.013 mmol/min mg protein) (Fig. 3b).

### λ-Carrageenan induces protein biosynthesis and the production of phenolic compounds in banana plants

An increase in total protein content was observed in 250 ppm λ-carrageenan treatment (1.89 ± 0.09 μg/g FW), 750 ppm treatment (2.51 ± 0.12 μg/g FW) and 1500 ppm treatment (2.48 ± 0.02 μg/g FW), compared to control (1.76 ± 0.04 μg/g FW) (Fig. 3c). Synchronously, an increase in total soluble phenol was detected in banana plants treated with λ-carrageenan, but a decrease occurred beyond 750 ppm treatment (Fig. 3d). From Fig. 3d, banana plants treated with 250 ppm and 750 ppm λ-carrageenan exhibited higher soluble phenol contents (2.49 ± 0.05 mg/g FW and 2.75 ± 0.05 mg/g FW, respectively), as compared to the control (2.31 ± 0.15 mg/g FW) and 1500 ppm treatment (2.35 ± 0.07 mg/g FW).

### λ-Carrageenan alters levels of H_2_O_2_, MDA, and ROS-scavenging enzymes in banana plants

As shown in Fig. 3e, banana plants treated with 1500 ppm λ-carrageenan treatment recorded the highest content of H_2_O_2_ (0.0230 ± 0.0009 μM/g FW). The banana plants treated with 250 ppm and 750 ppm of λ-carrageenan showed lower H_2_O_2_ contents (0.0133 ± 0.0003 μM/g FW and 0.0128 ± 0.0008 μM/g FW, respectively), as compared to control plants (0.0208 ± 0.0019 μM/g FW). Moreover, the pattern of increment in MDA level was similar to that in H_2_O_2_ content (Fig. 3f), where in 1500 ppm treatment was the highest (0.96 ± 0.10 nmol/g FW), followed by control (0.74 ± 0.08 nmol/g FW), 250 ppm treatment (0.48 ± 0.05 nmol/g FW) and the lowest was 750 ppm (0.42 ± 0.06 nmol/g FW).

On the contrary, the highest catalase content was detected in banana plants treated with 750 ppm of λ-carrageenan (0.195 ± 0.012 unit/mg protein), while the lowest content was observed in 1500 ppm of λ-carrageenan treatment (0.024 ± 0.001 unit/mg protein) (Fig. 3g). There was no significant difference (p > 0.05) between control plants (0.101 ± 0.017 unit/mg protein) and 250 ppm of λ-carrageenan treated plants (0.122 ± 0.007 unit/mg protein). Furthermore, an increase in peroxidase content was detected in all banana plants treated with λ-carrageenan (Fig. 3h). As shown in Fig. 3h, banana plants treated with 750 ppm λ-carrageenan had the highest peroxidase content (4.42 ± 0.76 units/mg protein), followed by 1500 ppm (2.78 ± 0.63 units/mg protein), 250 ppm (1.88 ± 0.27 units/mg protein), and control (0.93 ± 0.19 unit/mg protein).

### Nutrient uptake of banana plants in response to λ-carrageenan treatment

To determine the effect of λ-carrageenan on nutrient uptake in banana plants, macronutrients including nitrogen (N), phosphorus (P), potassium (K), calcium (Ca), magnesium (Mg); and micronutrients such as iron (Fe), manganese (Mn), zinc (Zn) were detected using ICP-OES system. Our result showed that banana plants treated with 750 ppm λ-carrageenan contained higher concentrations of N, P, K, Mg and lower concentrations of Ca, Fe, Mn, Zn as compared to the control (Table 1). Significant increments were recorded in 750 ppm treatment for N (0.389 ± 0.01 g/100 g), P (432 ± 11.36 mg/kg), K (343 ± 11.27 mg/100 g) and Mg (74.1 ± 1.24 mg/100 g), whereas P (574 ± 10.82 mg/kg), Ca (94.7 ± 1.45 mg/100 g), Mg (62.6 ± 2.29 mg/100 g) and Mn (77.1 ± 1.75 mg/kg) were significantly increased in 1500 ppm treatment. On the other hand, a significant decrement was observed in bananas treated with 750 ppm λ-carrageenan for Mn (11.0 ± 1.28 mg/kg). Likewise, a significant decrement in K content was recorded in 1500 ppm treatment (245 ± 14.93 mg/100 g) as compared to the control.

**Table 1:** Nutrient ions content of banana plants treated with different λ-carrageenan concentrations.

Nutrient content								
Concentration (ppm)	N (g/100 g)	P (mg/kg)	K (mg/100 g)	Ca (mg/100 g)	Mg (mg/100 g)	Fe (mg/kg)	Mn (mg/kg)	Zn (mg/kg)
0	0.365 ± 0.01	414 ± 6.24	315 ± 7.21	48.1 ± 7.15	40.1 ± 2.69	6.47 ± 0.43	60.9 ± 1.99	2.17 ± 0.62
750	0.389 ± 0.01*	432 ± 11.36*	343 ± 11.27*	36.3 ± 3.56	74.1 ± 1.24*	5.22 ± 0.70	11.0 ± 1.28*	1.08 ± 0.35
1500	0.337 ± 0.02	574 ± 10.82*	245 ± 14.93*	94.7 ± 1.45*	62.6 ± 2.29*	6.39 ± 0.10	77.1 ± 1.75*	1.31 ± 0.27

Means ± standard error followed by * in a row which indicates statistical significance between control and λ-carrageenan-treated samples (*P* < 0.05).

## Discussion

In the past few decades, food insecurity has always been a pressing issue in consequence of the growing human population. This issue has led to a surge in synthetic fertilizer usage in agricultural crop production^8^. However, extensive use of synthetic fertilizer had caused numerous health issues and environmental pollution. Thus, the development of an eco-friendly fertilizing approach is of the essence to enhance crop productivity and ensure food security. Carrageenan can be a potential environment-friendly plant growth promoter because it is a natural polymer extract from seaweed. Despite the application of carrageenan as plant elicitor has been reported in some plant species, little knowledge is known on the underlying mechanism of λ-carrageenan in promoting plant growth. In the present study, we investigated how different doses of λ-carrageenan could affect banana plants growth and what were the potential plant growth enhancement mechanisms effected by λ-carrageenan. We first determined the optimum concentration of λ-carrageenan applied to banana plants. The application of 750 ppm λ-carrageenan gave the optimum growth performance on banana plants with enhanced plant height, root length, pseudostem diameter, and fresh weight. These results are similar to previous reports in which foliar spraying of oligo-carrageenans stimulated plant growth in height, foliar biomass, and the number of leaves^13,14,20^. Additionally, González et al.^16^ also found that plant height and trunk diameter were enhanced via spraying with oligo-carrageenans. On the contrary, a higher concentration of λ-carrageenan (1500 ppm) caused stress or harmful effects on the banana plants as exhibited by the yellowing of leaves and stunted plant growth (Fig. 1). Based on the results obtained, the effect of λ-carrageenan is concentration-dependent. Our results are in congruent with previous studies suggesting that different concentrations of carrageenan stimulate or inhibit plant growth differentially^20,21^.

External stimuli from plant elicitors may affect plant growth and development by altering gene expression and physiological processes^22^. In this study, we found that the application of optimum λ-carrageenan potentially enhanced photosynthesis in banana plants as demonstrated by increased chlorophyll content, rubisco activity, and the expression of *cao* and *rbcL* genes in banana plants. Chlorophyll is essential for photosynthesis activity by harvesting light and generating energy^23^. Low chlorophyll content can directly limit photosynthesis, thus affecting plant growth. Rubisco is the key enzyme responsible for carbon fixation, where it fixes atmospheric CO_2_ into organic carbon within the Calvin cycle^23^. This will lead to glucose production, which is needed for plant growth. The *cao* gene plays a role in the chlorophyll metabolic pathway to convert chlorophyll *a* to chlorophyll *b*^24^. Meanwhile, *rbcL* gene is encoded for a major component of ribulose bisphosphate carboxylase, where it takes part in the primary event in carbon dioxide fixation namely carboxylation of D-ribulose 1, 5-bisphosphate^25^. Studies have shown that increase in total chlorophyll content is correlated with an increase in net photosynthesis^17,26^. The stimulatory effects of rubisco activity and chlorophyll content by λ-carrageenan could enhance the photosynthetic capacity in banana plants, which is shown in this study.

In addition, λ-carrageenan promoted plant growth in banana plants by inducing protein biosynthesis and the production of phenolic compounds. Our result revealed that optimum λ-carrageenan treatment increased total protein content and expression of *sams* gene. An increase in protein content may induce biosynthesis of secondary metabolites, which leads to plant growth stimulation^13,27^. Kok et al.^28^ also reported a similar positive correlation between protein content and plant growth. Moreover, *sams* gene plays a crucial role in synthesizing S-adenosylmethionine, a universal methyl group donor involved in transmethylation reactions for metabolism and plant development regulation, and also acts as a precursor in the biosynthesis of methionine, polyamines, nicotianamine and ethylene in plants^29^. In general, methionine is involved in protein synthesis whereas polyamines, nicotianamine, and ethylene are responsible for the regulation of plant development and homeostasis as well as stress tolerance. Previous reports had revealed that knockdown of *sams* caused stunted growth and asymmetric leaves in Chinese cabbage^30^, and dwarfism in rice^31^. On the other hand, the total phenol content was recorded higher in optimum λ-carrageenan treatment as compared to other treatments. At the molecular level, the expression of *tcm* gene was also highly upregulated in optimum treatment. Plant phenolic compounds play important role in regulatory signal pathway and modulating essential physiological processes, which are essential for plant growth^32^. As reported previously, plant phenolic compounds exert plant growth stimulation and regulation^32,33^. Moreover, tcm is responsible for phenolic biosynthesis, whereby it hydroxylates cinnamic acid at the C4 position to form *p*-coumaric acid^34^. The *p*-coumaric acid is an intermediate of the phenylpropanoid pathway, crucial for the production of various phenolic compounds such as flavonoids, tannins, and lignin^34,35^. In short, optimum λ-carrageenan treatment increased protein content and phenolic compounds in banana plants that stimulated plant growth.

Generation and accumulation of reactive oxygen species (ROS) such as hydrogen peroxide (H_2_O_2_), hydroxyl radical, and superoxide radical, in response to abiotic stresses, is highly reactive and toxic to biomolecules including proteins, lipids, and nucleic acids. This may lead to a state of oxidative stress in plant cells, affecting various physiological activities such as membrane disruption via lipid peroxidation and protein denaturation, as well as stunting growth^28,36^. In normal conditions, a low level of ROS is maintained in plants to ensure the regulation of the redox-signalling pathway^36^. Besides, excessive ROS in plant cells stimulate lipid peroxidation in cell membranes and increases the production of malondialdehyde (MDA). This MDA will further damage the cell membrane and eventually leads to cell death^37^. Hence, to minimize oxidative damage in plants, ROS-scavenging enzymes such as catalase and peroxidase will be produced to eliminate ROS and maintain homeostasis in plant cells^36–38^. In the present study, the application of optimum λ-carrageenan was shown to reduce H_2_O_2_ content, MDA content, and expression of *prx* gene in banana plants. On the other hand, expression of *cat* gene and contents of catalase and peroxidase were recorded higher in optimum treatment as compared to control. These results revealed that λ-carrageenan at optimum concentration may induce ROS-scavenging enzyme production to remove the effect of H_2_O_2_ accumulation and lipid peroxidation in plants, in order to overcome stress and sustain plant development. The ROS molecules are degraded via enzymatic reactions exerted by ROS-scavenging enzymes where H_2_O_2_ will be degraded into water and oxygen^38^. Reduction in ROS accumulation reduces oxidative damage in plants; therefore, the occurrence of lipid peroxidation may also be reduced. For example, an increase in enzymatic activities of peroxidase and catalase along with decrease in H_2_O_2_ generation and lipid peroxidation were observed in strawberries after blue light treatment^37^ and alfalfa after NaCl treatment^38^. Therefore, the optimum concentration of λ-carrageenan potentially strengthens the ROS scavenging system and free radical elimination capabilities in banana plants. Nonetheless, λ-carrageenan at higher concentrations may also activate the production of some ROS-scavenging enzymes and induce stress in plants at the same time. The high expression level of *prx* gene and contents of H_2_O_2_ and MDA were recorded in treatment with a higher concentration of λ-carrageenan. Upregulation of *prx* gene expression, which is involved in ROS metabolism, may induce the generation of H_2_O_2_ in plants and hence induce oxidative damage and lipid peroxidation^39^. Our results also showed that higher λ-carrageenan decreased the activity of catalase and increased the activity of peroxidase in banana plants. The decrease in catalase activity may lead to further accumulation of H_2_O_2_ in plants. Meanwhile, the massive H_2_O_2_ accumulation is unlikely to be fully offset by the reduction effect of peroxidase. For instance, oxidative stress was detected in rice leaves treated with excess iron although it showed no changes in catalase activity but increased activity of ascorbate peroxidase^40^. Growth stunting and yellowing of leaves were also observed in banana plants with the application of higher concentrations. This result is consistent with previously reported studies, whereby excessive ROS accumulation resulted in growth retardation, senescence, and programmed cell death^36,38^. Therefore, treatment with higher λ-carrageenan induces stress in banana plants via accumulation of H_2_O_2_, lipid peroxidation and reduced catalase activity.

Mineral nutrients are one of the important factors influencing plant growth and development. Nutrients are taken up by roots from the soil and transported throughout the plants for life processes^41^. Plants require large amounts of macronutrients and trace amounts of micronutrients to maintain their growth and development. Deficiency in nutrients may cause stunted growth, chlorosis, and even plant death. However, excessive nutrients could also pose toxic impacts to the plants such as generation of oxidative stress, damage to plant cells, and inhibit plant growth^42^. Our results showed that most of the macronutrients (N, P, K, and Mg) were significantly increased in banana plants treated with optimum λ-carrageenan. This was believed to be due to the enhancement of root structures induced by optimum λ-carrageenan. Previous reports stated that changing root structure may affect the capacity of plants to absorb nutrients from soil^43,44^. The enhanced root structure could facilitate soil exploration and offer more root surfaces for nutrient absorption, thus improving nutrient uptake in banana plants. Nitrogen, phosphorus, and potassium are the most important mineral elements for plants. Nitrogen is essential for protein synthesis, chlorophyll production, and regulation of cell division^45^. Similarly, phosphorus plays a vital role in photosynthesis, protein synthesis, and carbohydrate metabolism^46^. Potassium is mostly needed for several cellular processes such as the regulation of water and enzyme activities^47^. Whilst magnesium acts as a major component of chlorophyll involved in photosynthesis processes^41^. Deficiencies of these elements caused stunted growth in maize^45^, and reduced leaf area in cotton and soybean^47^. However, Mn was observed with significantly decreased in optimum λ-carrageenan treatment. The reduction in manganese may be caused by the high level of phosphorus in plants. Previously reported by Pedas et al.^46^ that a negative interaction between phosphorus and manganese had resulted in a decline in manganese uptake upon application of phosphorus in barley.

Nonetheless, λ-carrageenan at higher concentration may stimulate absorption of Ca and Mn. Massive amount of H_2_O_2_ was reported to accumulate in banana plants treated with higher concentration of λ-carrageenan. The H_2_O_2_ signalling may induce Ca^2+^ receptors and mediated Ca^2+^ influx in banana plants^48^. A high level of Ca^2+^ in plant cells will further trigger H_2_O_2_ generation, which may cause oxidative stress and damage to the plants. Similarly, Mn, as a micronutrient, is only required in a small amount for plant growth and development. Excessive Mn concentrations in banana plants will alter various metabolic processes and induce oxidative stress, resulting in disruption of cell homeostasis and eventually inhibiting plant growth^49^. Moreover, depletion of K was also detected in banana plants treated with a higher concentration of λ-carrageenan, where K deficiency may cause chlorosis and growth retardation^47^. Altogether, λ-carrageenan at optimum concentration increased uptake of macronutrients in banana plants and stimulated plant growth. However, a higher concentration of λ-carrageenan induced micronutrients uptake, imposing stress and toxicity in banana plants.

## Conclusions

**Figure 4:**
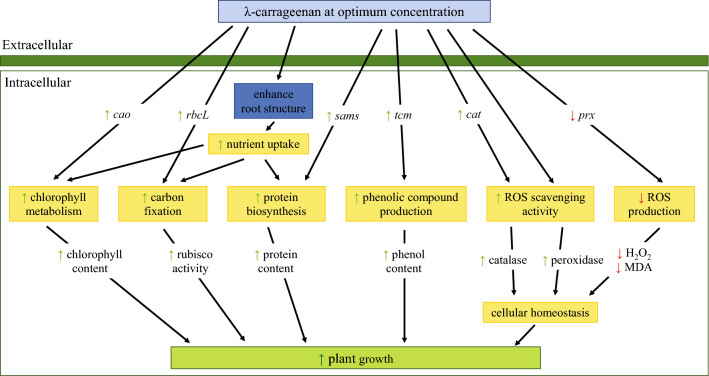
Proposed model demonstrating the mechanism of λ-carrageenan in enhancing plant growth in banana. Key: *cao*, *chlorophyllide a oxygenase*; *rbcL*, *ribulose-1,5-bisphosphate carboxylase*; *sams*, *S-adenosylmethionine synthase*; *tcm*, *trans-cinnamate 4-monooxygenase*; *cat*, *catalase*; *prx*, class III *peroxidase*; ROS, reactive oxygen species; rubisco, ribulose-1,5-bisphosphate carboxylase/oxygenase; H_2_O_2_, hydrogen peroxide; MDA, malondialdehyde; ↑ increased; ↓ decreased.

In this study, the different effects of λ-carrageenan on treated banana plants showed a dose-dependent response. λ-carrageenan at optimum concentration (750 ppm) potentially enhanced the growth of bananas via the increase in carbon fixation, protein biosynthesis, production of phenolic compounds, nutrient uptake, and maintenance of cellular homeostasis (Fig. 4). However, λ-carrageenan at higher concentration (> 750 ppm) induced stress response in banana plants with the observation of yellowing leaves as well as accumulation of hydrogen peroxide. Therefore, it is plausible that the application of λ-carrageenan at optimum concentration could be employed as promoter of growth in banana. This may lead to the development of an environmentally friendly fertilizer to increase agricultural crop production and sustain food security. However, further research should be devoted to the potential effects of λ-carrageenan in eliciting plant defense response against pathogens in banana plants and the evaluation of the potential of λ-carrageenan as a dual-functional bio-fertilizer in banana plants under field conditions.

## Methods

### Plant material and growth conditions

The two-month-old potted plantlets of *M. acuminata* cv. Berangan were obtained from the Global Green Tissue Culture Nursery (Klang, Malaysia). All plantlets were grown in polyethylene bags containing 250 g compost soil under long-day conditions (16 h of light and 8 h of dark; 70 μmol/m^2^ s photon flux density) at 25 ± 2 °C. All plantlets were irrigated three times a week for 2-week acclimatisation prior to treatment. All the experiments were performed in accordance with relevant guidelines and regulations.

### Plant treatment and growth measurement

The λ-carrageenan (22049, Sigma Aldrich, Germany) was prepared as stock solution at 10 mg/mL. Plantlets were treated with λ-carrageenan at 250 ppm, 750 ppm, and 1500 ppm respectively. Control plantlets were treated with distilled water. Treatment was applied twice via soil drench with an interval of one week, which are at the 1st day of treatment and the 8th day of treatment. Growth parameters such as plant height, root length, pseudostem diameter, and fresh weight were measured a day before treatment and a week after treatment. Leaf samples were collected from three biological replicates for each treatment and kept at − 80 °C. The experiment was repeated three times under identical experimental conditions.

### Analysis of gene expression in banana

Evaluation of gene expression in banana plants was performed by quantitative Reverse Transcription PCR (qRT-PCR). Total RNA was isolated from leaves of banana using the RNeasy Plant Mini Kit (Qiagen, Germany) according to manufacturer’s protocol. Leaf samples were homogenised in liquid nitrogen using a mortar and pestle. The concentration and purity of extracted RNA were determined using spectrophotometry. Reverse transcription was performed with the QuantiNova Reverse Transcription Kit (Qiagen, Germany) according to the manufacturer’s instructions. The resulting cDNA was analysed by qRT-PCR using QuantiNova SYBR Green PCR Kit (Qiagen, Germany) in Bio-rad CFX 96™ thermal cycler (Bio-Rad, USA) following the protocol described by Kamarudin et al.^50^. The housekeeping *glyceraldehyde 3-phosphate dehydrogenase* (*gapdh*) and *ubiquitin* (*ubq*) genes were used as reference genes^51^. The data was analysed using Bio-rad CFX Manager™ software. Gene expression levels were determined using the 2^−∆∆CT^ method^52^. Three technical and three biological replicates were analysed for each treatment. All the primers used for qRT-PCR are listed in Supplementary Table S1.

### Determination of chlorophyll content

Chlorophyll a, chlorophyll b and total chlorophyll contents were determined using the method described by Fortunato et al.^53^. Briefly, 200 mg of leaves were ground with a mortar and pestle in 1 mg/mL calcium carbonate. The extract was homogenised with 80% (v/v) acetone in the dark for 1 min at room temperature. The homogenate was filtered with Whatman paper, and the residues were washed four times with 80% (v/v) acetone. Readings were taken at 470 nm, 647 nm, and 663 nm via a UV/Vis spectrophotometer (Jenway, UK). Total chlorophyll content was estimated according to the equation proposed by Lichtenthaler^54^.

### Detection of rubisco activity

For determination of rubisco activity as reported by Khan et al.^55^, 1 g of leaves was homogenised in an extraction buffer containing 0.25 M Tris–HCl (pH 7.8), 0.05 M MgCl_2_, 0.0025 M EDTA and 37.5 mg DTT. The homogenate was centrifuged at 10,000 × g for 10 min at 4 °C. The corresponding supernatant was added to the reaction mixture containing 100 mM Tris–HCl (pH 8.0), 40 mM NaHCO_3_, 10 mM MgCl_2_, 0.2 mM NaDH, 4 mM ATP, 0.2 mM EDTA, 5 mM DTT, 1 U glyceraldehyde-3-phosphodehydrogenase, 1 U 3-phosphoglyceratekinase and 0.2 mM ribulose-1,5-bisphosphate. The absorbance was spectrophotometrically measured at a wavelength of 340 nm. Three biological replicates for each treatment were used and the experiment was repeated three times.

### Determination of total protein content

Total protein content was determined using the Bradford method as detailed by Bradford^56^. Approximately 150 mg of leaves were ground into a fine powder using a mortar and pestle and homogenised with 10 mM potassium phosphate buffer (pH 7.0) containing 4% (w/v) polyvinylpyrrolidone (PVP)^57^. After centrifugation at 12,000 × g for 30 min at 4 °C, the resulting supernatant was added to the Bradford reagent and incubated for 5 min. Absorbance was read at 595 nm and expressed as μg/g FW by comparing against the bovine serum albumin standard curve (0.2–1.0 mg/mL).

### Determination of phenolic content

Following a protocol modified from Dallagnol et al.^58^, the total phenolic content was measured using Folin-Ciocalteu reagent. Around 100 mg of leaves were homogenised in 80% (v/v) methanol and incubated overnight in darkness with shaking. The homogenate was centrifuged at 12,000 × g for 5 min. The corresponding supernatant was added to 0.25 N Folin-Ciocalteu reagent, 1 M sodium carbonate and distilled water. After incubation for 1 h at room temperature, the assay mixture was measured at 725 nm.

### Detection of hydrogen peroxide

Accumulation of hydrogen peroxide (H_2_O_2_) level in bananas was quantified using the protocol reported by Velikova et al.^59^ with a minor modification. Approximately 200 mg of leaves were homogenised in 2 mL of 0.1% (w/v) trichloroacetic acid (TCA) in an ice bath. The homogenate was centrifuged at 12,000 × g at 4 °C for 15 min. The resulting supernatant was added to 0.5 mL of 10 mM potassium phosphate buffer (pH 7) and 1 mL of 1 M potassium iodide. Absorbance was measured at 390 nm using a UV/Vis spectrophotometer (Jenway, UK) and expressed as µM/g FW through comparison against a hydrogen peroxide standard curve (25–100 µM/mL).

### Detection of malondialdehyde

Levels of malondialdehyde (MDA) were determined according to Kok et al.^60^. Briefly, 200 mg of leaves were homogenised in 10 mL of 10% (w/v) TCA. After centrifugation at 12,000 × g for 15 min, the supernatant was added to an equal volume of 0.6% (w/v) TBA in 10% (w/v) TCA. The reaction mixture was incubated in a water bath for 20 min at 100 °C. After cooling, the mixture was centrifuged at 12,000 × g for 10 min. The absorbance of the supernatant was read at 532, 600, and 450 nm. The MDA content was calculated using the formula: MDA content (μM) = 6.45 (OD_532_–OD_600_)–0.56 (OD_450_).

### Assay of catalase activity

Detection of catalase activity was assayed as described by Velikova et al.^59^. The reaction mixture consisted of 10 mM potassium phosphate buffer (pH 7.0), 35 μL H_2_O_2_ (3%) and 100 μL of crude enzyme. Absorbance was measured at 240 nm. Changes in absorbance were calculated and expressed as unit/mg protein.

### Assay of peroxidase activity

Peroxidase activity was determined by monitoring the consumption of hydrogen peroxide in a UV/Vis spectrophotometer at 420 nm over 3 min^53^. In brief, 250 mg of leaves was homogenised in 2 mL of 100 mM potassium phosphate buffer (pH 6.8) containing 1 mM EDTA, 1 mM PMSF, and 300 mg PVPP. The homogenate was centrifuged at 12,000 × g for 15 min at 4 °C. The supernatant (100 μL) was added to the assay mixture (3 mL) containing 100 mM potassium phosphate buffer (pH 6.8), 100 mM pyrogallol, and 100 mM H_2_O_2_. The peroxidase activity was expressed as unit/mg protein.

### Inductively coupled plasma optical emission spectroscopy (ICP-OES)

For nutrient analysis, leaf samples were harvested and dried at 65 °C for 72 h. The dried samples were ground to a fine powder and added to hydrochloric acid (HCl) and nitric acid (HNO3) (4:1). The samples were then digested in a microwave oven (CEM Mathews, NC, USA). The digested samples were left to cooled and diluted with distilled water. Determination of nitrogen was carried out by Kjeldahl method (AOAC 991.20)^61^. Determination of other mineral ions (P, Ca, K, Mg, Fe, Mn, and Zn) was carried out using PerkinElmer Avio 500 ICP-OES system as outlined in USEPA Method 6010D^62^.

### Statistical analysis

Results were analysed using GraphPad Prism 8.0.1 software (GraphPad Software, San Diego). Significant differences were determined using one-way analysis of variance (ANOVA) followed by Dunnett multiple comparison tests (T). Differences between mean values were statistically significant at a probability of 5% (P < 0.05). Standard errors were calculated for all mean values.

## Supplementary Information

Supplementary Table S1.

## Data Availability

All data generated or analysed during this study are included in this published article and its Supplementary Information file.
