# Supplementary material for: λ-Carrageenan promotes plant growth in banana via enhancement of cellular metabolism, nutrient uptake, and cellular homeostasis
Source: Sci Rep. 2022 Nov 16;12:19639. doi: 10.1038/s41598-022-21909-7 (PMC9669011; doi:10.1038/s41598-022-21909-7)
Supplement: Supplementary file 1 — Supplementary Table S1. [file 41598_2022_21909_MOESM1_ESM.pdf]

Article type: Original Research

Title: λ-carrageenan promotes plant growth in banana via enhancement of cellular metabolism, nutrient uptake, and cellular homeostasis

Kah-Lok Thye[1], Wan Muhamad Asrul Nizam Wan Abdullah[1], Zetty Norhana Balia Yusof[2], Chien-Yeong Wee[3], Janna Ong-Abdullah[1], Jiun-Yan Loh[4], Wan-Hee Cheng[5], Dhilia Udie Lamasudin[1,*], & Kok-Song Lai[6,*]

1 Department of Cell and Molecular Biology, Faculty of Biotechnology and Biomolecular Sciences, Universiti Putra Malaysia, 43400 Serdang, Selangor, Malaysia; kahlok94@gmail.com (K.L.T.); wanmuhamadasrul@gmail.com (W.M.A.N.W.A.); janna@upm.edu.my (J.O.A.); dhilia@upm.edu.my (D.U.L.)

2 Department of Biochemistry, Faculty of Biotechnology and Biomolecular Sciences, Universiti Putra Malaysia, 43400 Serdang, Selangor, Malaysia; zettynorhana@upm.edu.my (Z.N.B.Y.)

3 Biotechnology and Nanotechnology Research Centre, Malaysian Agricultural Research and Development Institute, 43400 Serdang, Selangor, Malaysia; cywee@mardi.gov.my (C.Y.W.)

4 Centre of Research for Advanced Aquaculture (CORAA), UCSI University, 56000 Cheras, Kuala Lumpur, Malaysia; lohjy@ucsiuniversity.edu.my (J.Y.L.)

5 Faculty of Health and Life Sciences, INTI International University, Persiaran Perdana BBN, Putra Nilai, 71800 Nilai, Negeri Sembilan, Malaysia; wanhee.cheng@newinti.edu.my (W.H.C.)

[6] Health Sciences Division, Abu Dhabi Women's College, Higher Colleges of Technology, 41012 Abu Dhabi, United Arab Emirates; lkoksong@hct.ac.ae (K.S.L.)

*Corresponding authors: dhilia@upm.edu.my (D.U.L.); lkoksong@hct.ac.ae (K.S.L.)

Supplementary Information

Supplementary Table S1. Primers used in qRT-PCR.

**Target genes**	**Sense**	**Sequence (5'-3')**
Chlorophyllide a oxygenase	Forward	GCAAGAAGGAATGGTTTGGA
	Reverse	TATCCAAAAGAAGCCCATGC
Ribulose-1,5-bisphosphate carboxylase	Forward	ACAAAGGGCGATGCTACCAC
	Reverse	TGGGAATTCGCAGATCCTCC
S-adenosylmethionine synthase	Forward	GGACTGCTGCTCGATGTTGA
	Reverse	ATCACCACCAAGGCCAATGT
Trans-cinnamate 4-monooxygenase	Forward	TGTCGAAGCTGTGTGGCAAG
	Reverse	GGAAGATTTCGCCGAAGCG
Class III *peroxidase*	Forward	CAGACGAGATAGCAAGGAAAGCCAC
	Reverse	TCCCACGAGGTTGACACAG
Catalase	Forward	GGCGTCAACACCTACACCTT
	Reverse	AGCTTCCACTCCGGGTAGTT
**Housekeeping genes**	**Sense**	**Sequence (5'-3')**
Glyceraldehyde 3-phosphate dehydrogenase	Forward	CCAGCAAGGATGCCCCAATGT
	Reverse	CTGCACAACCAACTGTCTTGCT
Ubiquitin	Forward	TCCAGCGGCTCCAAGTTCTC
	Reverse	GCGAGCGTTTGGTCGTCATTC
